# Prevalence of supplement use in recreationally active Kazakhstan university students

**DOI:** 10.1186/s12970-018-0220-4

**Published:** 2018-04-10

**Authors:** Denis Vinnikov, Zhanna Romanova, Anar Dushpanova, Karashash Absatarova, Zhazira Utepbergenova

**Affiliations:** Al-Farabi Kazakh National University, al-Farabi avenue 71, Almaty, Kazakhstan 050040

**Keywords:** Physical activity, Cross-sectional, Swimming

## Abstract

**Background:**

Little is known about the supplements use and recreational sport practices in Kazakhstan university students. Therefore, the aim of this study was to ascertain supplements use prevalence and their predictors in this population.

**Methods:**

Cross-sectional survey of both undergraduate and graduate level students was completed in 2017 et al.-Farabi Kazakh National University, the largest higher institution in the country, from almost all Schools. A 45-item questionnaire was used to record physical activity, supplements use, lifestyle attributes (smoking, alcohol, sleep, etc.) and eating habits, and adjusted regression models were used to verify predictors of supplements use.

**Results:**

Of the entire sample of 889 students (70% females), 526 (59%) were practicing recreational physical activity (RPA), and walking, jogging and track and field was the most popular activity type (38%). *N* = 151 (29%) students reported the use of any supplement (31% in men and 27% in women), whereas the most popular supplement type were vitamins. Supplement use was most prevalent in swimmers (55%). Age (odds ratio (OR) 1.19 (95% confidence interval (CI) 1.04–1.37), use of fitness tracker (OR 6.26 (95% CI 3.90–10.03)) and low-fat diet (OR 1.95 (95% CI 1.23–3.10)), but not income predicted supplements use in adjusted models.

**Conclusions:**

With more than half of students exercising regularly, only less than one-third use supplements with a very strong association with fitness tracker use.

## Background

Supplement use is a common attribute in those involved both in recreational sport and in elite athletes. Data from meta-analyses of numerous studies report different prevalence data with regard to a particular supplement, whereas vitamins tend to be reported most, especially in no-elite athletes. Supplements use profiles are extensively described in professional sportsmen, including runners, footballers and other sports [1–3]. Also, given high supplement use prevalence in the military and armed forces, the use is quite well described in these populations. Thus, recent meta-analysis in the military showed that up to 71% of women in Marine Corps were using supplements [4].

Supplement use has also been characterized in adolescents and young adults, and in these groups, vitamins were reported the most common type of supplements, whereas individual sport as opposed to team sport, university level as opposed to high school were associated with greater supplement use prevalence [5]. With all these studies, it is still unclear what other predictors of supplement use exist in the university community, given the wide interplay of individual and social determinants, including even muscle dysmorphia and orthorexia [6]. In adolescents, any supplement use prevalence is somewhat around 50% [7, 8], whereas the data are very limited in the university students. Greater academic load, financial challenges of new life away from parents, peer pressure on alcohol and smoking may affect the attitudes of students to recreational physical activity and associated supplement use.

In Central Asia, a part of the former Soviet Union, with a total population of 71 million people from five countries, behavioral traits and physical activity attributes are underrepresented in the literature; no reports exist today to characterize the patterns of supplements use in the university students from the region. Given that the overall supplement use prevalence, as well as the predictors of their use with regard to specific recreational physical activity in the university students from the region are not known, and financial constraints may drive both recreational physical activity and supplements use, we hypothesized that supplements use will have a strong association with income in students. Therefore, the aim of this study was to ascertain supplements use prevalence and their predictors in a group of Kazakhstan university students.

## Methods

This is a cross-sectional analysis of physical activity and supplements use in Kazakhstan university students in a representative sample of al-Farabi Kazakh National University in Almaty. The study was approved by a Committee on Bioethics of the School of Public Health et al.-Farabi Kazakh National University, and each participant provided a written informed consent to participate. We invited students to participate through the internal university media and during presentation in classes. All volunteering students signed an informed consent to participate. They were offered a 45-item self-administered questionnaire in Russian in their off-class time. Al-Farabi Kazakh National University is a leading and the largest higher education facility in the country, offering tuition in a wide range of sciences, including School of Public Health, School of Chemistry, School of Physics, School of Biology, School of Biotechnology, School of Political Science, School of Mathematics, and few other. Both undergraduate (years 1–4) and graduate students (years 5–6) were enrolled. From a total of approx. 14,000 students, 889 agreed to participate and provided their filled questionnaires.

We collected data on demographics (age, sex, year of study) with living conditions questions (residence at the dorm, parent, or rent); cumulative monthly income in tenge (local currency, one USD is approximately 325 tenge); questions on tobacco products and alcohol use, including waterpipe and smokeless tobacco use. Self-reported smoking status was classified into never-, ever- and daily smokers, also asking for a number of smoked cigarettes a day and smoking duration. In an alcohol section, we clarified preferred beverage.

We then asked about adherence to daily routine (waking and going to bed at one time daily; trying to do that but not always good with that; often different time of sleep; and failure to fulfill sleeping recommendations and staying awake at night). Sleep duration was also categorized into (8 h or more a day; 6–8 h a day; and less than 6 h); with an adjacent question on the level of daytime sleepiness. Physical activity was classified using series of questions adapted from Health-Promoting Lifestyle Profile II, adult version. We asked if a student walked 6 km or 10,000 steps a day including weekends; was engaged in any leisure physical activity (recreational physical activity (RPA)) at least 3 times a week for at least 40 min. This question stratified students into those involved in RPA and those who are not. Those involved in RPA should have attributed them to one of the leading activity from the list offered: (1) walking, jogging or track and field; (2) cycling; (3) swimming; (4) volleyball, basketball, soccer or other games using a ball; (5) Step aerobics, fitness or dancing; (6) yoga; (7) gym, weight- or powerlifting; (8) martial arts (combat sport); and (9) all other.

We also addressed the motivation for RPA in students asking them to choose one of the most relevant options: (1) RPA is part of my healthy lifestyle; (2) losing or maintaining lower weight; (3) gaining weight or muscle mass; (4) RPA improves my mood; (5) I am coping with stress; (6) I am moving towards professional career in sports; and (7) I am trying to make new friends. Supplement users were identified whether they answered yes to the question “Are you currently using any of the sport supplements (nutrients used to attain better results in RPA)?”. Those who did, were directed to the next question asking to choose one of the most often used supplements of the list offered: (1) vitamins or multivitamins; (2) creatine, energy drink or pre-workout mixture; (3) protein; (4) carbohydrate and protein mix or gainer; (5) amino acids, including branched-chain amino acids (BCAA); (6) fat burners; (6) carnitine or arginine; and (7) *Tribulus terrestis* extract.

In addition, we asked whether a student used any electronic fitness tracker for the recreational physical activity. The next six questions were adapted from Health-Promoting Lifestyle Profile II to ascertain nutrition habits of the university students. We asked whether responders (1) choose a diet low in fat, saturated fat, and cholesterol; (2) limit use of sugars and food containing sugar (sweets); (3) eat 2–4 servings of fruit each day; (4) eat 3–5 servings of vegetables each day; (5) eat 2–3 servings of milk, yogurt or cheese each day; (6) eat breakfast daily.

The primary outcome in this analysis was supplement use, treated as a binary variable. Secondary outcomes were cigarette and waterpipe smoking, electronic cigarette, smokeless tobacco use, alcohol use, use of fitness tracker, different attributes of sleep; and nutrition habits (eating pattern). For each continuous variable, we report mean with its standard deviation, whether the data were normally distributed, otherwise – median with its interquartile range (IQR). We tested difference in variance between two groups using Mann-Whitney U-test, but for more groups we report *p*-values of F-statistic from ANOVA. Binary variables were compared using contingency tables with Fischer test.

After descriptive statistics in univariate analyses, we tested selected demographic and lifestyle attributes and variables against supplement use, expressed as a binary variable. These crude analyses are reported with significance values of selected predictors. Significant variables (*p* < 0.05) from such analysis were then considered predictors in multivariate regression models, in which their association with supplement use first in crude and then in adjusted models. We set up two types of adjusted regression models, including (1) adjusted for basic significant demographic confounders, such as sex, age and income; (2) adjusted for sex, age, income and all other predictors for a specific model, which predicted supplement use in the univariate analysis with *p* < 0.05. From these analyses, we report odds ratios (OR) with their corresponding 95% confidence intervals (CI). All tests were performed in NCSS 12 (Utah, USA).

## Results

The sample of students in this study (*N* = 889) were mostly females, living in the dormitories and having an income below 50,000 tenge a month. 59% of all students were practicing RPA, with the number much greater in male students.

Five-hundred and twenty six students reported regular physical activity. 202 students (38%) reported walking, jogging or track and field; 20 (4%) were cycling; 38 (7%) were swimming; 90 (17%) were regularly playing with a ball (basketball, etc.); 61 (12%) were doing step-aerobics, fitness or dancing; 12 (3%) were practicing yoga; 54 (10%) were attending gym to do weights; 33 (6%) were doing martial arts, and the remaining 3% were doing other sports. Of those exercising regularly (*N* = 526), 151 (29%) reported the use of any sport supplement. 61 (31%) of all regularly exercising men (*N* = 194) and 90 (27%) of all regularly exercising women (*N* = 332) reported regular use of sport supplements. 118 (78%) used only one supplement, whereas 33 (22%) used two or more supplements. Their prevalence was almost equally distributed when types of activities were compared and ranged from 35% in cycling to 55% in swimming (Table 1). Vitamins were most often used supplements in the surveyed students. The least popular supplements in students were carnitine and arginine, except in those lifting weights in the gym; however its use prevalence was as low as 7% in this group. None of the regularly exercising students were taking *Tribulus terrestis*.

**Table 1:** The prevalence of supplements use stratified by activity type in a sample of exercising students

	Any supplement	Vitamins	Energy booster	Protein	Weight gainer	Amino acids, BCAA	Fat burners	Carnitine or arginine	*Tribulus terrestis*
Walking, jogging, track and field	47%	35%	6%	4%	2%	3%	1%	0%	0%
Cycling	35%	25%	0%	5%	0%	10%	0%	0%	0%
Swimming	55%	24%	11%	8%	8%	18%	0%	0%	0%
Volleyball, basketball, etc.	37%	20%	8%	9%	9%	6%	1%	1%	0%
Step aerobics, fitness, dancing	44%	18%	5%	8%	13%	2%	5%	0%	0%
Yoga	42%	17%	8%	8%	8%	8%	0%	0%	0%
Weightlifting, gym	48%	22%	6%	7%	9%	7%	4%	7%	0%
Martial arts	45%	15%	3%	18%	6%	12%	3%	0%	0%

In the univariate analysis of predictors of supplements use, we found that older students, those with higher income, smoking cigarettes or using electronic cigarettes or smokeless tobacco, as well as those using any kind of fitness tracker and sleeping 8 h as recommended were more likely to use supplements as part of their exercising plan (Table 2). Of note, the leading motivation to exercise was also a strong determinant of supplements use, and moreover, sport as part of healthy lifestyle significantly decreased the likelihood of supplements use, as opposed to weight management. Those losing or maintaining lower body weight had the greatest number of supplements users.

**Table 2:** Univariate comparison of selected predictors between supplement users with their non-using counterparts

	Supplement users	Supplement non-users	p
N (%)	151 (29)	375 (71)	–
Male sex, N (%)	61 (40)	133 (35)	0.32
Age, years	20.3 ± 1.8	19.8 ± 1.4	0.004
Living with parents, N (%)	13 (9)	48 (13)	0.228
Income 51,000 tenge and more	45 (30)	81 (22)	0.055
Income 101,000 tenge and more	20 (13)	16 (4)	0.001
Daily smoking, N (%)	24 (16)	35 (9)	0.046
Waterpipe ever smoking, N (%)	55 (36)	119 (32)	0.307
Electronic cigarette regular use, N (%)	24 (16)	18 (5)	0.001
Snuss ever use, N (%)	24 (16)	19 (5)	0.001
Never alcohol drinking, N (%)	93 (62)	262 (70)	0.083
Strictly following daily routine, N (%)	65 (43)	96 (26)	0.001
Sleep 8 h and more daily, N (%)	63 (42)	99 (26)	0.001
No daytime sleepiness, N (%)	69 (46)	99 (26)	0.001
Use of fitness tracker, N (%)	78 (52)	44 (12)	0.001
Low fat and cholesterol diet, N (%)	107 (71)	192 (51)	0.001
Limited sugar, cakes and soda diet, N (%)	103 (68)	167 (45)	0.001
Daily fruit consumption, N (%)	107 (71)	202 (54)	0.001
Daily vegetables consumption, N (%)	105 (70)	198 (53)	0.001
Daily diary products consumption, N (%)	93 (62)	175 (47)	0.002
Individual goal in sport			
Part of my healthy lifestyle	7 (5)	168 (45)	0.001
Losing or maintaining lower weight	82 (54)	89 (24)	0.001
Gaining weight or muscle mass	13 (9)	26 (7)	0.581
Improving my mood	12 (8)	38 (10)	0.513
Coping with stress	5 (3)	10 (3)	0.773
Moving towards professional sport career	7 (5)	10 (3)	0.278
Making new friends	7 (5)	2 (1)	0.003

We then tested whether the selected variables predicted the use of supplements, trying to derive the strongest predictor of their use in students in both crude and adjusted regression models. We found that some of the variables exhibited quite strong collinearity, such as all variables of healthy eating. All three variables of healthier sleep were also correlating with each other. Therefore, we elected to include age, income, daily smoking, strict daily routine, sleep 8 h daily, use of a fitness tracker, low fat and cholesterol diet, exercising for healthy lifestyle and exercising to lose or maintain weight as predictors in adjusted models. As outcomes, we chose any supplement use, vitamin use, energy booster or creatine use, and protein-gainer-BCAA use. Table 3 shows that age, use of fitness tracker and low fat diet, but not income or sleep routine predicted the use of supplements in general. The use of fitness tracker was strongly associated with the use of supplements. We failed to find any predictors of creatine or energy booster use, and even adding powerlifting or goal:need to gain weight still could not predict this outcome. In addition, the only significant predictor of protein/gainer/BCAA use was the use of fitness tracker. Adding powerlifting alone or powerlifting with the goal:need to gain weight did not change the overall R^2^ of this model, whereas these two extra predictors were non-significant.

**Table 3:** Logistic adjusted regression models for four selected outcomes of supplements use

Predictor	Outcome			
	Any supplement use Model R2 = 0.31	Vitamin use Model R2 = 0.27	Energy booster or creatine use Model R2 = 0.09	Protein-gainer-BCAA use Model R2 = 0.07
Age	1.19 (1.04–1.37)	NS	NS	NS
Income 101,000 tenge and more	NS	NS	NS	NS
Daily smoking	NS	NS	NS	NS
Strict daily routine	NS	NS	NS	NS
Sleep 8 h daily	NS	2.18 (1.17–4.09)	NS	NS
Use of fitness tracker	6.26 (3.90–10.03)	10.66 (5.85–19.43)	NS	2.73 (1.52–4.87)
Low fat and cholesterol diet	1.95 (1.23–3.10)	NS	NS	NS
Goal: healthy life style	NS	2.37 (1.15–4.91)	NS	NS
Goal: lose or maintain weight	NS	NS	NS	NS

Note: data are presented as adjusted OR with 95% CI. Selected variables were not included in some models. All included variables are adjusted for each other. *NS* – non-significant

## Discussion

To our best knowledge, this is the first study from Central Asia on the practices of supplements use in recreational sport. We found that almost one third of those exercising regularly, use any supplement for different reasons. Vitamins were most often used supplements, whereas swimming was the type of activity with the highest prevalence of any supplement use. We also found that those who exercising regularly for their healthy life style, report the lowest supplements use prevalence. In the sample of university students, we found that the use of fitness tracker was the strongest predictor of supplements use, which had the greatest association with vitamins; however the OR was still very strong for any supplements use. Moreover, vitamins use had also some significant association with other healthy lifestyle self-reported components, such as sleep 8 h a day and healthy lifestyle as a self-reported goal of activity. The data we obtained will reject our initial research hypothesis that supplements use is mostly associated with income.

The findings of this study in a sample of university students were somewhat surprising. Although higher income was associated with the likelihood of supplements use, the association became non-significant in an adjusted regression model. Fitness tracker use was the strongest predictor not only for selected supplements, but for any supplement use history, which should direct further supplement marketing to the need to address this association. We were also quite surprised to see that the prevalence of supplement use in recreational swimming was higher than in weightlifting, as we were with the protein, weight gainers and BCAA more prevalent use in those doing martial arts than fitness of weightlifting. We believe that university students may be prone to quite often shift of specific recreational sport activity to another, resulting from some peer pressure or alternating academic workload. Therefore, in such population, recreational sport activity may unlikely have a long set history, where the students try themselves in different types with time.

While supplement use and practices were extensively studied in young athletes with known prevalence [2, 3, 7–9], the latter remains unclear in the university students and no data exist today from any Central Asian country. Evidence exists for the efficacy of a few supplements in use, like creatine, beta-alanine, bicarbonate, caffeine, nitrate/beetroot juice and, perhaps, phosphate [10], however, marketing and advertisement of many other widely used supplements are still directed to specific populations, including young people of the university age. Supplements’ prevalence in our study was somewhat similar to the one amongst students in other countries. In the sample of Italian high school and university students, 37% students had ever taken supplements [5], whereas in another study of volunteer runners, their use prevalence was 28% [11], as in the current report. In addition, supplements use prevalence in our presentation was surprisingly lower in those students who exercised as part of their perceived healthy lifestyle, which likely represented specific pattern of beliefs among Kazakhstan students that dietary supplements are not part of exercising plan, when exercising for healthy lifestyle.

One of most pronounced findings of this study was a strong association of supplements use with an electronic fitness tracker use, irrespective of the socioeconomic status. From our perspective, this may be indicative of the complex interaction of beliefs of recreational physical activity with the use of modern technology of effective exercising. The implications of such interactions could be the association of supplements use with the use of modern technology in exercising in marketing. With a very fast and widespread use of electronic fitness trackers in young people, such as university students, supplements prevalence may tend to grow in future.

This study was limited to the university students only, which may be a limitation in generalizing the study findings to young people of the similar age, given academic pressure and the need to live away from parents with some financial concerns. Unequal distribution of sexes in this analysis may also underestimate the true supplements use prevalence in males. Another limitation of this analysis is a relatively small sample size for this type of study. We also need to mention there was no objective verification of data self-reported by the students, which can obviously generate some exposure misclassification bias. Of note, very few, if any, studies of this kind will use self-report data verification techniques. One of the ways to confirm physical activity could be the use of electronic activity trackers, however other prevalence data in the literature still rely more on questionnaires. Finally, we could not study beliefs and attitudes to the supplements use in the current cohort, which could have subjectively predicted their use and prevalence, which we consider another limitation of this analysis.

## Conclusions

In conclusion, this is the first analysis of the supplements use practices from Central Asia, which showed that almost one-third of regularly exercising university students, use any kind of sport supplements, with the likely greater prevalence in swimming. Vitamins were the commonest supplements in use, associated with healthy lifestyle as a primary goal of exercising regularly. With regard to almost all supplement categories, the use of an electronic fitness tracker had a strong association with supplements use.

## Abbreviations

BCAA: Branched-chain amino acids
CI: Confidence interval
IQR: Interquartile range
OR: Odds ratio
RPA: Recreational physical activity
